# Transcription Factor AP4 Mediates Cell Fate Decisions: To Divide, Age, or Die

**DOI:** 10.3390/cancers13040676

**Published:** 2021-02-08

**Authors:** Matthew Man-Kin Wong, Sancy Mary Joyson, Heiko Hermeking, Sung Kay Chiu

**Affiliations:** 1 Department of Cellular and Molecular Medicine, University of Ottawa, Ottawa, ON K1H 8M5, Canada; mwong043@uottawa.ca; 2 Sprott Center for Stem Cell Research, Ottawa Hospital Research Institute, Ottawa, ON K1H 8L6, Canada; 3 Department of Biological Sciences, Xi’an Jiaotong-Liverpool University, Suzhou 215123, China; S.Joyson@liverpool.ac.uk; 4 Cellular and Molecular Physiology, Institute of Translational Medicine, University of Liverpool, Liverpool L69 3BX, UK; 5 Experimental and Molecular Pathology, Institute of Pathology, Ludwig-Maximilians-University Munich, 80337 Munich, Germany; 6 German Cancer Consortium (DKTK), Partner site Munich, 80336 Munich, Germany; 7 German Cancer Research Center (DKFZ), 69120 Heidelberg, Germany; 8 Faculty of Health and Life Sciences, University of Liverpool, Brownlow Hill, Liverpool L69 7TX, UK

**Keywords:** transcription factor AP4, cell proliferation, apoptosis, EMT, tumorigenesis

## Abstract

**Simple Summary:**

Here, we review the literature on Activating Enhancer-Binding Protein 4 (AP4)/transcription factor AP4 (TFAP4) function and regulation and its role in cancer. Elevated expression of AP4 was detected in tumors of various organs and is associated with poor patient survival. AP4 is encoded by a Myc target gene and mediates cell fate decisions by regulating multiple processes, such as cell proliferation, epithelial-mesenchymal transition, stemness, apoptosis, and cellular senescence. Thereby, AP4 may be critical for tumor initiation and progression. In this review article, we summarize published evidence showing how AP4 functions as a transcriptional activator and repressor of a plethora of direct target genes in various physiological and pathological conditions. We also highlight the complex interactions of AP4 with c-Myc, N-Myc, p53, lncRNAs, and miRNAs in feed-back loops, which control AP4 levels and mediate AP4 functions. In the future, a better understanding of AP4 may contribute to improved prognosis and therapy of cancer.

**Abstract:**

Activating Enhancer-Binding Protein 4 (AP4)/transcription factor AP4 (TFAP4) is a basic-helix-loop-helix-leucine-zipper transcription factor that was first identified as a protein bound to SV40 promoters more than 30 years ago. Almost 15 years later, AP4 was characterized as a target of the c-Myc transcription factor, which is the product of a prototypic oncogene that is activated in the majority of tumors. Interestingly, AP4 seems to represent a central hub downstream of c-Myc and N-Myc that mediates some of their functions, such as proliferation and epithelial-mesenchymal transition (EMT). Elevated AP4 expression is associated with progression of cancer and poor patient prognosis in multiple tumor types. Deletion of *AP4* in mice points to roles of AP4 in the control of stemness, tumor initiation and adaptive immunity. Interestingly, ex vivo AP4 inactivation results in increased DNA damage, senescence, and apoptosis, which may be caused by defective cell cycle progression. Here, we will summarize the roles of AP4 as a transcriptional repressor and activator of target genes and the contribution of protein and non-coding RNAs encoded by these genes, in regulating the above mentioned processes. In addition, proteins interacting with or regulating AP4 and the cellular signaling pathways altered after AP4 dysregulation in tumor cells will be discussed.

Transcription factors (TFs) regulate gene expression by binding to specific cis-regulatory elements (CREs) around their target genes and recruit other DNA binding regulatory factors to form complexes that mediate combinatorial effects on transcription [1]. About three decades ago, the ubiquitous transcription factor activator protein 4 (TFAP4 or AP4) along with AP1 was found to activate Simian virus 40 (SV40) late transcription by binding to the E-box motif, 5′-CAGCTG-3′, in a synergistic manner [2]. In a genome-wide ChIP-Seq analysis, we found that AP4 mainly binds to the 5′-CAGCTG-3′ motif, but may also bind to the related transcription factor SNAIL binding motif, 5′-CACCTG-3′, albeit with a lower frequency [3]. In terms of molecular structure, AP4 has an N-terminally located basic region, which mediates DNA binding, and a helix-loop-helix (bHLH) and two leucine-zipper (LZ) regions that mediate dimerization. AP4 exclusively forms homodimers. The two LZ regions were found to prevent heterodimerization with other bHLH-LZ or bHLH proteins and thereby ensure AP4 homodimer formation [4].

As the AP4 binding site is only six nucleotides long, there are theoretically 732,421 AP4 binding sites (once every 4096 bps) in the human genome. However, it is clear that not every site is bound by AP4. In order to comprehensively study the global AP4 occupancy and identify genes controlled by AP4, we applied a combination of microarray, chromatin immunoprecipitation, and next generation sequencing analyses after ectopic AP4 expression [3]. Thereby, we found that 1458 genes out of ~27,000 genes are occupied by AP4 and significantly, differentially regulated in the colorectal cancer cell line DLD-1. Presumably, some of these genes are regulated by AP4 in a cell-type specific manner. The upregulated and repressed genes cover a wide range of functions including cell proliferation, apoptosis, stemness, differentiation, metabolism, epithelial-mesenchymal transition (EMT), and metastasis.

## 1. AP4-Mediated Repression vs. Induction of Target Genes

Although AP4 was originally identified as a transcriptional activator for viral gene expression [2], many subsequent studies showed that it functions as a repressor of transcription for many genes. In the literature, there are several studies demonstrating an activating function of AP4 in the development of mammals. Badinga et al. showed that AP4 binds to the E-box motifs at the insulin-like growth factor binding protein-2 (IGFBP-2) promoter and acts as a trans-activator in luciferase reporter assay [5]. Another gene that is transactivated by AP4 is the dopamine β-hydroxylase gene (*DBH*), which is involved in the conversion of dopamine to norepinephrine in neural crest stem cells [6]. It was shown that the promoter of the *DBH* gene contains both AP4 and SP1 binding motifs within its promoter region. Although not binding to the promoter by itself, the transcription factor GATA-3 interacts with either AP4 or SP1 bound on the *DBH* promoter to facilitate gene activation. Interestingly, AP4 also binds to the promoter of the pancreatic amylase 2A gene, and DNase footprinting experiments showed that there are several AP1 binding sites near the AP4 binding motif. However, the authors did not investigate whether the binding of AP4 changes the expression of amylase 2A [7]. We also identified an enrichment of the AP1 and SP1 binding sites in the vicinity of promoters occupied by AP4 by using in silico methods and the AP4 ChIP-Seq results obtained in DLD-1 cells described above [3]. Whether the enrichment of AP1 and SP1 binding sites is linked to activation or repression by AP4 or whether it occurs in a cell-type specific manner is currently unknown.

In the aforementioned genome-wide analysis of genes regulated by AP4 in the colorectal cancer cell line DLD-1 [3], we found that the prevalence of AP4 acting as a repressor is higher than mediating gene activation. A total of 884 direct targets of AP4 were identified, and 530 of those were significantly down-regulated 24 h after ectopic AP4 expression, whereas 354 were induced by more than 1.5 fold. In addition, promoters repressed by AP4 showed an increased number of AP4 binding sites and were located closer to the transcriptional start site when compared to AP4-induced genes. As a result, AP4-repressed genes displayed stronger ChIP-Seq peaks at their promoter regions than activated genes. The differential regulation of target genes may be determined and mediated by numerous AP4-associated proteins, such as chromatin-histone deacetylases (HDACs), SWI/SNF-related, matrix associated, actin-dependent regulator of chromatin (SMARCs) proteins, and histone methyl-transferases (EHMTs) that were identified in a proteomic approach by Chen and our lab [8].

Notably, AP4 was shown to suppress gene transcription in many studies that focused on single AP4 target genes. For example, in human immunodeficiency virus (HIV-1) latently infected cells, AP4, along with histone deacetylase 1 (HDAC1), negatively regulates viral gene expression by binding to the HIV-1 long terminal repeats (LTR) within the viral promoter, and preventing access of the TATA-binding protein (TBP; TFIID) to the TATA-box [9]. Similarly, AP4 downregulates the transcription of the human papillomavirus type-16 *E7* oncogene, which is required for the maintenance of the transformed phenotype, by binding to the P542 promoter [10]. In a screen for p53 activating factors, AP4 was identified as a repressor of the human homolog of the murine double minute 2 (*HDM2*) gene [11]. We could show, that the Glutamine and proline Q/P-rich acidic domain of AP4 is required to interact with numerous accessory proteins, such as HDAC1 and SP1, and thereby facilitates repression of the *HDM2* gene [8].

Moreover, AP4 forms a protein complex with transcription corepressor geminin (Gem) to repress the temporal expression of the neuronal gene phytanoyl-CoA α-hydroxylase-associated protein 1 (PAHX-AP1) in the fetal brain. The AP4-Gem complex recruits silencing mediator of retinoic acid and thyroid hormone receptors (SMRT) and histone deacetylase 3 (HDAC3) to down-regulate the expression of neuron-specific genes in non-neuronal cells. During further development of the adult brain, expression of both AP4 and Gem declines, leading to higher expression of PAHX-AP1. Dual-specificity tyrosine-phosphorylation regulated kinase 1A (DYRK1A), which is involved in neurodevelopment, is repressed by the AP4-Gem complex, but, in the fetal brain of patients with Down syndrome, there is a decline in the expression of this complex, resulting in the transcription of the *DYRK1A* gene [12]. Furthermore, a decrease in the human angiotensinogen promoter transcriptional activity was observed when AP4 was overexpressed in an in vitro CAT assay [13]. This indicates that AP4 may repress the human angiotensinogen gene, which codes for the precursor of the vasoconstrictor hormone angiotensin-II in controlling blood pressure [14]. Many additional, genes repressed by AP4 were identified in the genome-wide study mentioned above (3): among them were *CDH1*, *CD44*, *CLDN1*, *4,7, GDF15*, and *OCLN*.

The repression of AP4 target genes is achieved, at least in part, via the recruitment of chromatin-modifying enzymes. For example, AP4 recruits HDACs for transcriptional repression of HIV-1 [9] and HDM2 [8]. However, treatment with HDAC inhibitors is not sufficient to mitigate AP4-mediated repression of the *HDM2* gene. Using the *HDM2*-P2 promoter DNA sequences containing AP4 binding site “CAGCTG” to pull down AP4 and its interacting proteins, we identified the chromatin-remodeling SWI/SNF complexes and histone H3K9 methyltransferases GLP/G9A (EHMT1 and EHMT2), in addition to the components of the HDAC complex [8]. These data imply that AP4 recruits multiple chromatin-modifying complexes for repression of its target genes. Since AP4 can act as both trans-activator and repressor, it remains to be determined whether the AP4 protein interactome changes in these two modes and what is the underlying mechanism that allows AP4 to switch between mediating induction versus repression at different target genes. One point to be mentioned is that the interpretation as to how AP4 controls normal developmental functions from chromatin-based assays should be cautious because immortalized and tumor cell lines may have altered chromatin states and the transcription factor occupancy may not reflect that of normal cells.

## 2. AP4 DNA Binding Sites as Targets for SNPs

Single-nucleotide polymorphisms (SNPs) are positions in the genome having single nucleotide variations that are present at a frequency of higher than 1% in the human population [15]. Many of the SNPs are linked to diseases. It is believed that single nucleotide changes in the genome may alter the binding of transcription factors and, hence, the expression of genes, which leads to the onset of diseases. Indeed, about one-third of the SNPs identified in genome-wide association studies (GWAS) overlap with a transcription factor binding site [16]. Previous studies showed that AP4 displays differential binding towards specific SNPs. For example, enrichment of AP4 is detected at the interleukin 2 receptor alpha (*IL2RA*) SNP rs12722522*C, which is associated with type 1 diabetes [17]. Another example is the SNP rs1800734 located at the promoter of the mismatch repair gene homolog 1 (*MLH1*) gene. Loss of *MLH1* leads to impaired DNA mismatch repair (MMR), which causes microsatellite instability in some cancer types [18,19]. The binding of AP4 to the *MLH1* promoter protects the region from DNA methylation at least in part via the enrichment of the zinc finger repressor protein CTCF [20]. Of note, a significant difference in the CTCF ChIP signal between the mutated and wild-type allele is not detected upstream of the rs1800734 allele at the position of a predicted CTCF binding site. Instead, CTCF is only enriched near the wild-type rs1800734 allele which is occupied by AP4. This observation correlates well with our previous finding that AP4 interacts with CTCF via its acidic region [8]. Interestingly, the rs1800734 G > A mutation disrupts the AP4 binding sequence, CAGCTG, located at the promoter of *MLH1*. The loss of AP4 binding leads to an increase in DNA methylation at the promoter region and the epigenetic silencing of *MLH1* [21]. In another study, Liu and colleagues found that the rs1800734 G > A mutation in several colorectal cancer cell lines enhances the long-range interactions between rs1800734 and the promoter and 3′ UTR region of the doublecortin-like kinase 3 gene (*DCLK3*), resulting in an increase in the expression of this potential oncogene [22]. The authors reasoned that the rs1800734 G > A mutation disrupts the AP4 binding site and creates a binding motif for the E26 transformation-specific (ETS) transcription factor and thereby mediates activation of *DCLK3*.

## 3. AP4 Controls Cell Proliferation, Senescence, and Apoptosis

**Figure 1:**
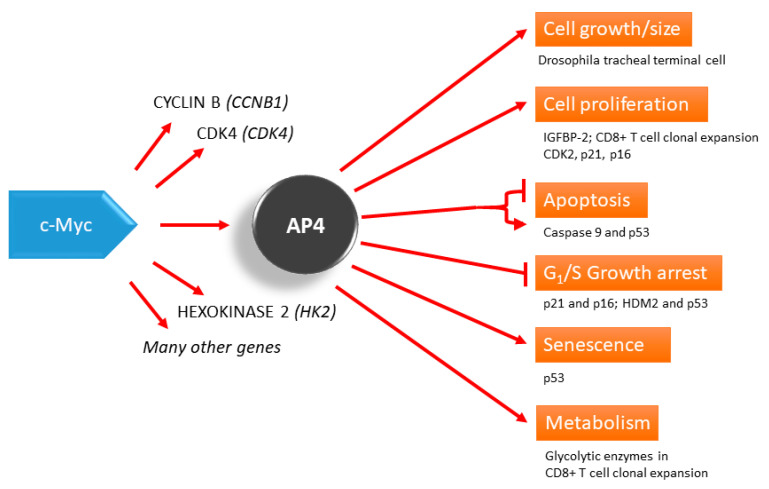
AP4 as a mediator of the c-Myc function. AP4 is involved in many processes, such as the control of cell proliferation, which may be a part of developmental programs, such as CD8+ T cell clonal expansion and *Drosophila* tracheal terminal cell branching. AP4 can also regulate apoptosis, growth arrest, and senescence, in which p53 seems to be an important downstream player. Evidence from siRNA knockdown experiments shows that AP4 represses apoptosis.

Mitogens interact with receptors presented on the cell surface and activate signaling pathways related to cell proliferation, survival and death [23]. Activation of the c-Myc gene represents a nodal point for most mitogenic signaling pathways. Activated c-Myc regulates numerous transcriptional programs that translate the incoming signals into cellular responses necessary for cell proliferation, such as protein and RNA synthesis, cell cycle activation and activation of glycolysis. Overstimulation of the mitogenic signaling pathways due to mutations can lead to the activation of cell cycle inhibitors, like p16 and p21, which may mediate cell cycle arrest or permanent arrest/senescence [24]. c-Myc, as well as other members in the Myc family of proteins, are known to be involved in the development of vertebrates [25], and when the expression is derailed, c-Myc causes cancer formation [26,27]. c-Myc is known to be a transcriptional regulator of AP4 [28], and some of the c-Myc functions are executed via the activity of AP4 (Figure 1). Similarly, in *Drosophila*, d-Myc regulates AP4 homolog Cropped (*crp*) to control the development of the terminal branching of *Drosophila* tracheal tubes and cell size. In addition, overexpression of *crp* results in increased cell size and nuclear size [29], which may presumably be due to endoreplication, a process similar to the cell cycle without cytokinesis.

Among all human AP4 controlled genes, the cell cycle regulatory genes and the apoptosis genes were enriched in a Gene Ontology (GO) analysis, suggesting that AP4 regulates cell proliferation and death [3]. It was shown that human c-Myc, a driver of cell proliferation, up-regulates AP4 expression to repress the transcription of the cyclin-dependent kinase (CDK) inhibitors p21 and p16, which inhibit the cell-cycle, suggesting that a critical level of AP4 is needed for correct cell cycle maintenance or prevention of its inhibition [28,30]. Indeed, mouse embryo fibroblasts (MEFs) derived from mice with a deletion of *AP4* display premature senescence [30]. The effects of *AP4* deletion were dependent on the up-regulation p16 and p21 since RNAi-mediated repression of these factors was able to reduce senescence. In addition, *AP4*-deficient MEFs ectopically expressing c-Myc and mutant RAS were resistant to transformation and did not form tumors in mice. Interestingly, ectopic expression of AP4 combined with mutant RAS resulted in tumors in *p53*-deficient MEFs but not in *p53*-proficient, wild-type MEFs. Since the combination of c-Myc and RAS expression was sufficient for tumor formation in wild-type MEFs, c-Myc presumably has additional oncogenic properties that are not mediated by AP4.

AP4 was also required for cell-cycle re-entry after mitogenic re-stimulation of starved MEFs with 10% serum, since *AP4*-deficient MEFs showed a strong delay in DNA-replication in this assay [31]. Interestingly, activation of AP4-ER, a tamoxifen-inducible fusion of AP4 to the estrogen receptor (ER), was only able to induce S-phase entry and proliferation in the presence of 1% serum but not at 0.25% serum in human diploid fibroblasts (HDF) [31]. This was mediated by the direct activation of *Cyclin Dependent Kinase 2/CDK2* by AP4. However, activation of Myc-ER, is able to induce proliferation in low serum and requires AP4, since deletion of *AP4* blocked Myc-induced S-phase entry [31]. In addition, in other studies, cell cycle arrest or decrease in cell number were observed after inactivation of AP4 using siRNAs [32,33,34]. Therefore, AP4 appears to be required, but is not sufficient, for induction of cell cycle entry and proliferation by mitogenic stimulation and after c-MYC activation.

Taken together, a certain level of AP4 expression is required for efficient cell cycle progression, but elevated AP4 expression is not sufficient to stimulate cell proliferation in mammals. A role of AP4 in cell cycle control and maintenance is also suggested by the observation that *AP4*-deficient MEFs showed a delay of 24 h in the onset of DNA replication in this assay and a defect in cytokinesis after mitogenic stimulation [31].

In addition to its role in cell cycle regulation and proliferation, AP4 also mediates apoptosis of mouse lymphomas treated with dexamethasone by binding to the promoter region and the activation of the cell death signal inducer (pro)caspase-9 to initiate apoptosis [35]. Tadakuma and colleagues showed that AP4 is expressed at a particular physiological level to maintain the basic level of pro-caspase 9 in the cells to prepare for any stress challenges. However, when the level of AP4 is lowered by knockdown of AP4 using siRNA [32,33] or knockout of AP4 in MEFs [31], cells also undergo apoptosis. It is not clear whether the diverging results are due to the use of different cell types or treatments of the cells. In contrast, activation of AP4 (in the form of AP4-ER after the addition of hydroxytamoxifen) was shown to be ineffective in inducing apoptosis [31]. Therefore, stress, such as transfection, is hypothetically needed together with a high level of AP4 to induce apoptosis, as we observed apoptosis only in cells transiently transfected with AP4 but not in the cells stably transfected with AP4 and induced with doxycycline (our unpublished data and the example of AP4-ER activation mentioned above). Further studies are required to validate this hypothesis.

In transformed (HeLa and HCT116 cells), as well as non-transformed cells (retinal pigment epithelial cells), it was observed that AP4 expression is reduced in the G2 phase due to the β-TrCP-mediated ubiquitination and degradation [36]. When the non-degradable AP4 (E135A/S139A) mutant was expressed in HCT116 cells, nuclear atypia and chromosome segregation were observed, and, subsequently, DNA damage response was activated, implying that an elevated, deregulated level of AP4 may lead to aberrant cell division and genomic instability.

Interestingly, our recent research shows that confluent retinal pigment epithelium (RPE) cells display senescence only when AP4 is over-expressed for more than three weeks in addition to a slowdown of cell proliferation [37]. RPE cells are immortal cells exhibiting cell-cell contact inhibition and proper tight and gap junctions [38], which make the cells suitable for studying how cells respond to a long-term tumorigenic challenge. AP4 is known to induce p53 expression directly by binding to the *TP53* gene promoter to cause premature senescence [39]. In another study, we demonstrated that a consistently low level of ectopic c-Myc expression induces AP4-mediated senescence in post-confluent RPE cells [37]. However, an elevated and persistent expression level of c-Myc increases the number of apoptotic cells but decreases senescence. Using siRNA-mediated knockdown, AP4 was shown to be required for the c-Myc-induced senescence. At a higher c-Myc level, p53 is induced by both c-Myc and AP4 to initiate apoptosis rather than cellular senescence. This study suggests that the choice of whether a cell enters cell death or aging depends on the relative levels of c-Myc and AP4 [37].

## 4. AP4 in Adaptive Immunity

A sophisticated adaptive immune system is essential for antigen-specific, long-lasting protection against pathogens. The adaptive immune system consists of two parts, namely the cell-mediated and antibody-mediated immune responses. In the cell-mediated immune responses, activated antigen-specific T cells are generated to fight against foreign antigens, and correct temporal expression of CD4 and CD8 proteins is essential for cell-fate determination between CD4^+^ helper T-cells and CD8^+^ cytotoxic T cells [40,41]. Inside the thymus, the most immature T cells are CD4 and CD8 double negative (DN). After β-selection [41], CD4^+^ and CD8^+^ double positive (DP) cells are selected. DP T cells that express relevant T cell receptors are screened by positive selection and differentiate into CD4^+^ or CD8^+^ single positive (SP) cells [42,43]. Interestingly, AP4 was shown to affect the decision of T cells fate in part by mediating the down-regulation of CD4 synergistically with Runx proteins in T cells [44]. In DN T cells where CD4 expression is repressed, AP4 binds to the proximal enhancer of the *CD4* gene. Consistent with the previous finding that AP4 interacts with HDAC1 [8], HDACs are thought to be one of the mediators in the AP4-mediated repression of CD4, as histone H3K9 hyperacetylation is detected at the proximal enhancer of the *CD4* gene in AP4-deficient T cells [44].

Besides suppressing the expression of the *CD4* gene, AP4 is also essential for maintaining the optimal proliferation and proper development of CD8 SP T cells. In activated CD8 SP T cells, c-Myc is essential for the initial activation of AP4 expression. Chou et al. found that AP4 is dispensable for the initial proliferation of activated CD8 SP T cells within the first 3 days after a viral infection, when c-Myc is expressed at a high level [45]. From day 4 to day 6 after infection, when c-Myc starts to decline and AP4 is still expressed at a high level in wild-type CD8 SP T cells, AP4 knockout cells have a reduced proliferation rate and cell size compared to wild-type cells. Subsequent microarray and ChIP-seq experiments showed that more than half of the c-Myc and AP4 ChIP-seq peaks were overlapped, and about one-fourth of the differentially expressed genes in AP4-knockout T cells are the shared targets of c-Myc and AP4. Gene ontology analysis showed that these shared targets are related to metabolism, transcription, and several translational pathways [46]. Importantly, the majority of the shared binding sites are still bound by AP4 at 5 days after viral infection when c-Myc expression declines. These data indicate that AP4 is required for the activation of genes to sustain the metabolic needs of CD8 SP T cells after c-Myc expression declines. Of note, the expression of a stabilized form of c-Myc (c-Myc T58A) rescues the decrease in proliferation and cell size in AP4 knockout T cells, but it only partially rescues the decrease in the number of terminally differentiated KLRG1^+^ CD8^+^ cells [46]. This implies that AP4 has its unique functions in CD8+ cell differentiation. In future studies, it would be interesting to focus on genes that are bound by AP4 alone but not c-Myc.

In the antibody-mediated immune response, antibody-secreting plasma B cells and memory B cells are generated in the germinal centers (GC) inside the lymphoid organs [47]. GC are dynamic microenvironments for the selection and maturation of B cells [48]. In the dark zone (DZ) of GC, B cells proliferate rapidly and undergo somatic hypermutation to generate random mutations in the variable domains of the B cell receptor (BCR). The cells then migrate to the light zone (LZ), where they interact with CD4^+^ follicular helper T cells. The B cell clones expressing high-affinity of antigen-specific BCR will be selected and migrate back to the DZ for further rounds of proliferation and somatic hypermutation [49]. As one of the mediators of the cell cycle, AP4 was shown to be essential for the proliferation and development of high affinity B cell clones in the DZ. Induced by c-Myc, which is expressed only in the LZ, AP4 is expressed in both LZ and DZ, and its expression in DZ is maintained by IL-21 [45]. B cell-specific AP4 deletion in mice results in a decrease in GC B cell number and GC size. B cells expressing AP4 in the dark zone also are more actively dividing and undergo more rounds of somatic mutation. In addition, B cell-specific AP4-knockout mice show a compromised immune response, as they are less capable of clearing up the viral load in serum after lymphocytic choriomeningitis virus-mediated chronic infection. Among the 520 upregulated genes in AP4^+^ DZ B cells, the majority of these genes are related to the metabolic and protein translational pathways. This implies that, similar to the roles of AP4 in CD8^+^ T cells, AP4 expression in DZ B cells is necessary for maintaining the metabolism of the cells. Another similarity between AP4 expressed in B cells and CD8^+^ T cells is that their expression is, in both, maintained by interleukins. In CD8^+^ T cells, the expression of AP4 protein drops after the withdrawal of IL-2 [46]. Similarly, IL-21 increases the expression of AP4 in cultured B cells in a dose-dependent manner [45]. It is not clear how interleukins regulate the expression of AP4, but the regulation is presumably mediated via post-transcriptional mechanisms, as the expression of AP4 encoded from the retrovirus in CD8^+^ T cells is also IL-2 dependent [46].

## 5. Pathological Functions of AP4 in Cancer

As discussed above, AP4 is involved in a very diverse array of physiological functions, and, as expected, de-regulation of AP4 expression can have pathological consequences. The following is a brief discussion of the roles of deregulated AP4 in cancers.

**Figure 2:**
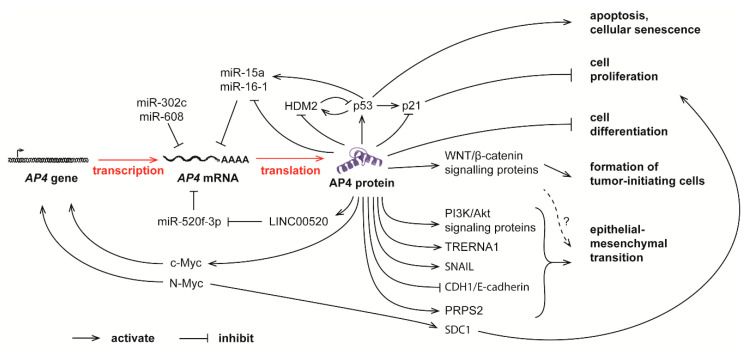
Complex feedback loops between AP4 and many other molecules in controlling normal physiological processes and tumor progression. The mRNA level of AP4 is controlled by c-Myc and N-Myc, as well as several miRNAs. Elevated AP4, in turn, promotes cell proliferation, apoptosis, senescence, tumor-initiating cell formation, and epithelial-mesenchymal transition (EMT).

Many recent studies have shown that AP4 is highly expressed in various types of tumor, including breast cancer [28], colorectal cancer [3,50], gastric cancer [32], pancreatic cancer [51], prostate cancer [52], non-small cell lung cancer [53], and hepatocellular carcinoma [54]. AP4 expression is an independent prognostic marker to predict the overall survival time [32,55,56] and cancer progression in terms of metastasis [3,52,57]. In addition, AP4 is essential for maintaining cell cycle progression of cancer cells [32,52,55] and regulates intestinal tumor initiation [58]. Figure 2 summarizes some of the known molecular functions of AP4 which will be discussed in detail below.

## 6. AP4 Regulates Epithelial-Mesenchymal Transition and Cell Migration in Cancer Cells

The epithelial-mesenchymal transition (EMT) is a process in which stationary epithelial cells lose their polarity and dissociate from neighboring tissues to become more motile and invasive mesenchymal-like cells. EMT is essential for early post-implantation development, embryogenesis, and tissue regeneration and wound healing after injury [59]. In later stages of cancer progression, EMT occurs in the initial steps of metastasis, in which tumor cells disseminate from their primary site, grow, and form tumors in other parts of the body, eventually leading to the death of patients [60,61].

Jackstadt et al. showed that AP4 represents an EMT-TF (EMT-inducing transcription factor), similar to SNAIL or ZEB1/2 [3]. In our global expression and DNA-binding analysis in the colorectal cancer cell line DLD-1, we found that AP4 binds to the promoters of and up-regulates many EMT effectors and marker genes, such as *SNAIL*, *Vimentin*, *FOS*, *LEF1*, and *N-cadherin*, and directly represses *CDH1/E-cadherin*. In addition, ectopic AP4 induced EMT and AP4 was necessary for c-Myc-induced EMT. In a xenograft mouse model, inactivation of AP4 prevented the formation of lung metastases after injection of CRC cell lines. Furthermore, elevated AP4 expression correlated with poor patient survival and increased distant metastases formation [3]. Using the metastatic breast cancer MDA-MB-231 cell line, our group showed that AP4 can activate cell migration and invasion by activating the mutant p53-R280K protein, and the expression of dominant-negative AP4, which lacks the DNA-binding domain, decreases cell mobility [62]. Furthermore, AP4 knockdown in colorectal cancer cells inhibits cell migration and invasion, and AP4 overexpression had the opposite effects [55].

AP4 is a direct target of N-Myc, which is highly expressed in approximately 25% of the tumors of neuroblastoma patients [63]. Correlated with the aforementioned roles of AP4 in cancer, AP4 knockdown in N-Myc-expressing neuroblastoma cells inhibits both cell migration and proliferation [64]. Similar to c-Myc, N-Myc binds to the E-boxes on the *AP4* gene to transactivate its expression. Interestingly, N-Myc and AP4 bind to some target genes simultaneously to regulate their expression. In neuroblastoma cells, both AP4 and N-Myc bind to the promoters of *SDC1* and *PRPS2* to regulate their expression. Syndecan-1, SDC1, is a transmembrane protein that is highly expressed in various cancer types [65] and is required for cell invasion and proliferation in myeloma and glioma cells [66,67]. Ribose-phosphate pyrophosphokinase 2, PRPS2, is an enzyme involved in nucleotide biosynthesis which is essential for tumorigenesis driven by c-Myc [68]. It is also needed for cell invasion and proliferation in colorectal cancer [69] and prostate cancer [70]. It remains to be determined whether AP4 regulates the expression of SDC1 and PRPS2 and plays a role in disease progression via these two genes in these cancer types.

## 7. AP4 Is Essential for Cell Cycle Regulation and Cellular Stress Response in Cancer

Besides mediating EMT in cancer, AP4 is also essential for maintaining cell cycle progression and preventing the cells from undergoing apoptosis. AP4 is required for c-Myc-mediated exit from cell cycle arrest in breast cancer cells [28], and siRNA-mediated knockdown of AP4 in gastric cancer cell lines leads to cell cycle arrest [32]. In glioma cells, the cell proliferation rate is positively correlated with the AP4 expression level, while the rate of apoptosis is negatively correlated with the AP4 level [71]. A similar correlation between AP4 expression levels and proliferation rate was also observed in colorectal cancer and prostate cancer cell lines [52,55]. In addition, downregulation of AP4 is required for microRNA-induced cell cycle arrest. In colorectal cancer cells, expression of *AP4* mRNA lacking its 3′-UTR, and thus resistant to microRNA degradation (is discussed in detail below), blocks miR-15a-induced G1 cell cycle arrest [72].

Emerging evidence shows that an optimal level of AP4 is required for the cells to survive through stress conditions in cancer cells. For example, AP4 knockdown in gastric cancer cells sensitizes the cells to anticancer drugs [73]. Furthermore, overexpression of AP4 in breast cancer cell line MCF7 sensitizes the cells to DNA damage-mediated cell death caused by etoposide [28]. In non-small cell lung cancer, the expression of miR-608 leads to downregulation of AP4, which sensitizes the cells to doxorubicin-induced apoptosis [53]. These results imply that AP4 may be a regulator of cellular stress responses in cancer, and further studies have to determine whether an optimal AP4 level is needed for cell survival through stress response in normal cells.

## 8. Role of AP4 in Stem Cells and Tumor Initiating Cells

Tumor-initiating cells (TICs), also known as cancer stem cells, are a subpopulation of cancer cells that have the ability to self-renew, differentiate, and give rise to tumors [74,75,76]. Studies have shown that TICs are resistant to various cancer therapies [75] and contribute to cancer relapse after chemo- and radiotherapy [77]. Importantly, TICs are enriched in metastatic tumors [78,79]. Since AP4 is a mediator of EMT, which drives metastasis, it is reasonable to anticipate that AP4 expression may induce the formation of TICs. Indeed, ChIP-Seq analyses revealed that AP4 binds to and induces the stemness marker genes *LGR5* and *CD44* in colorectal cancer cells [3]. Other stemness-related markers, such as CD133, NANOG, and SOX2, are upregulated in hepatocellular carcinoma cell lines HepG2 and HCC-LM3, overexpressing AP4 [54]. Overexpression of AP4 also enhances the tumorsphere forming ability, which is a characteristic of TICs when cultured in vitro. Similarly, AP4′s expression level is positively correlated with the percentage of the TIC-like side population in HepG2 cells. In another study, Boboila and colleagues identified AP4 as an important effector of N-Myc in a whole-genome shRNA library screen performed in neuroblastoma cells with inducible N-Myc expression. Silencing of AP4 induced neuronal outgrowth and cell differentiation, correlating with its role in mediating stemness in cancer cells [80]. Intestinal-epithelial cell specific deletion of *AP4* in mice resulted in a decrease in the number of intestinal stem cells and an increase in Paneth cells [58]. In addition, intestinal-epithelial cell specific loss of *AP4* in *Apc^min^* mice, a model for inherited colorectal cancer, decreased the number and size of intestinal adenomas and significantly increased the survival of mice [58]. Notably, tumor formation in *Apc^min^* mice is dependent on elevated c-Myc expression, since c-Myc is a target of the TCF/β-cat- complex, which is controlled by the WNT/APC pathway [81]. Therefore, these results suggest that AP4 is an important mediator of c-Myc-driven tumorigenesis. Intestinal organoids and tumoroids derived from *AP4*-deficient mice displayed a decrease in stemness. mRNA expression profiling suggested that the AP4 target genes are responsible for these effects are within the Notch and Wnt/β-catenin signaling pathways [58]. This study implies that AP4 promotes the formation of intestinal tumors via its role in stem cell formation. These promising findings suggest that AP4 is a potential therapeutic target to inhibit the formation and relapse in cancer.

In line with a central role of AP4 in stem cells, AP4 was recently shown to be an important component of a transcription factor network involved in the reprogramming of mouse fibroblast and hepatocytes and the generation of iPSCs (induced pluripotent stem cells) [82]. Papathanasiou et al. found that AP4 is part of a gene regulatory network composed of nine transcriptional regulators (9TR; Cbfa2t3, Gli2, Irf6, Nanog, Ovol1, Rcan1, Taf1c, Tead4, and AP4), which are directly targeted by the reprogramming factors Oct4, Sox2, Klf4, and c-MYC (OSKM) [82].

## 9. Connections between Non-Coding RNAs and AP4 in Cancer

Recent studies show that many non-coding RNAs (ncRNAs) are key regulators in developmental processes and diseases [83]. Relating the functions of ncRNA in cancer, three subtypes of ncRNAs are most well-studied: long non-coding RNAs (lncRNAs) which are longer than 200 nucleotides [84]; microRNA (miRNA) which are around 22 nucleotides in length [85], and circular RNAs (circRNAs) which are closed loops of RNA of 100 to 4 kb in length [86]. Dysregulation of various ncRNA and their pathological effects were previously reviewed [87,88]. Interestingly, the *AP4* mRNA is the target of several ncRNAs. Many studies showed that the 3′-UTR region of AP4 mRNA binds to various miRNAs (Figure 2). These include: miR-15a, miR-16-1 and miR-302c in colorectal cancer [57,72], miR-608 in non-small cell lung cancer [53], and miR-520f-3p in glioma cells [71]. In these studies, the expression level of the identified miRNAs inversely correlates with the expression level of AP4, demonstrating that the binding of ncRNA to the 3′-UTR of AP4 leads to the degradation of AP4 mRNA and inhibition of EMT. Notably, miR-15a/miR-16-1 represent tumor suppressive miRNAs, which are deleted in lymphomas [89]. In addition, these miRNAs are directly induced by the p53 tumor suppressor protein [90]. Therefore, the repression of AP4 may be relevant for tumor suppression by the p53/miR-15a/16-1 axis. In addition, AP4 binds to the promoters of the miR-15a and miR-16-1 host gene *DLEU2* and thereby represses miR-15a/miR-16-1 expression in colorectal cancer cells [72]. Therefore, AP4, miR-15a, and miR-16-1 form a negative feedback loop to regulate EMT, cell migration, invasion, and metastasis in colorectal cancer cells [72].

On the other hand, AP4 directly induces the expression of ncRNA genes by binding to their promoter regions. For example, in gastric cancer cells, AP4 binds to the promoter region of translation regulatory long non-coding RNA 1 (TRERNA1), transactivates its expression, and promotes TRERNA1-mediated cell migration and invasion [91]. Recently, it was found thatAP4 binds to the promoter region of LINC00520, a novel lncRNA, and transactivates its expression in glioma cells. LINC00520 competes with AP4 mRNA for the binding of miR-520f-3p, and the expression of LINC00520 protects AP4 mRNA from degradation. miR-520f-3p, AP4, and LINC00520 therefore also form a feedback loop to mediate cancer progression in glioma [71]. It remains to be determined whether AP4 also forms regulatory feedback loops with ncRNA in other types of cancer.

## 10. Pathways Involved in AP4-Mediated Regulation of EMT and the Cell Cycle in Cancer

The WNT/β-catenin signaling pathway is commonly dysregulated in cancer. The roles of WNT/β-catenin signaling in cancer were reviewed previously [92]. Interestingly, WNT/β-catenin signaling activation increases the oncosphere-forming capability of hepatocellular carcinoma cells [93]. To find the link between AP4, the WNT/β-catenin pathway, and TICs, Song and colleagues analyzed the RNA-seq data from hepatocellular carcinoma samples. They found that the abundance of AP4 correlates with the expression levels of the WNT/β-catenin signaling genes. Besides, AP4 binds to the promoter of the WNT/β-catenin signaling genes *DVL1* and *LEF1* and positively regulates their expression. Importantly, knockdown of DVL1 or LEF1 reduces the number of oncospheres formed and the percentage of TIC-like side population in AP4-overexpressing hepatocellular carcinoma cells [54], indicating that WNT/β-catenin signaling is essential for AP4-mediated enhancement of TIC formation.

More evidence comes to light that WNT/β-catenin signaling is important for EMT activation in cancer cells [94,95]. LEF1, the aforementioned downstream target of AP4, is a positive regulator of EMT [96,97]. However, further experiments are needed to confirm whether AP4 activates EMT via the WNT/β-catenin pathway. Another pathway that induces EMT is the PI3K/Akt pathway [98], which raises the possibility that AP4 activates EMT in cancer via PI3K/AKT signaling. Indeed, a recent study found that the AP4 expression level in hepatocellular carcinoma cell lines correlates positively with the level of phosphorylated AKT and its downstream targets. The addition of the PI3K inhibitor LY294002 blocks AP4-mediated activation of AKT and the subsequent increase in cell migration and invasion [56]. Taken together, these data suggest that AP4 mediates EMT, at least in part, through the PI3K/Akt pathway.

Multiple studies suggested that AP4 may control the expression of the genes within the HDM2-p53 pathway. Regulation of these genes is essential for processes, such as tumor suppression, DNA damage repair, and cellular senescence [99]. In normal somatic cells, HDM2 and p53 form a negative feedback loop [100]. In stress conditions, this feedback loop is interrupted, causing an up-regulation of p53 and subsequently of its downstream targets [101]. In colon cancer cell line HCT116, AP4 binds to the promoter of *HDM2* [8] and thereby inhibits its transcription [11]. In addition, p53 suppresses the expression of AP4 via miR-15a and miR-16-1 in colon cancer cell lines [72]. Interestingly, we previously showed that AP4 binds to the promoter of the *p53* gene to activate its expression in post-confluence retinal pigment epithelium (RPE) cells for cellular senescence [37]. We also demonstrated that AP4 acts upstream to regulate the expression of the mutant p53-R280K protein in the human breast cancer cell line MDA-MB-231, which implies that AP4 and p53 form a regulatory feedback loop [39,62]. However, further experiments are needed to confirm whether *p53* is a direct target of AP4 in tumor cells. AP4 also binds to the promoter of the *p21* gene, a downstream target of p53, and a mediator of cell fate under stress [102] to inhibit its expression [28]. In the MCF7 breast cancer cell line, c-Myc targets the first intron of the *AP4* gene and transactivates its expression [28]. Interestingly, AP4 can also bind to the promoter of *c-Myc*, and c-Myc’s expression is reduced after AP4 knockdown, which indicates that AP4 forms a regulatory loop with c-Myc in breast cancer cells [103]. Moreover, the cross talk of regulation between Myc and AP4/Crp was also found in a study of an activated Ras and loss of function of scribble in forming invasive tumors in *Drosophila* [104]. In this study, Crp/AP4 has been implicated as a key regulator of a tumor transcriptome in *Drosophila*. However, the in vivo knockdown experiments showed that AP4/Crp only has a limited role in tumor formation.

In summary, AP4 regulates the fate of cancer cells by forming regulatory networks with numerous signaling pathways, transcription factors, and ncRNAs, among them the Wnt and Notch pathways, c-Myc, p53, HDM2, miRNAs (miR-15a/16-1, miRNA-520f-3p), and lncRNAs (LINC00520, TRERNA1).

## 11. Conclusions

As discussed above, AP4 is involved in the decision to proliferate, to die, or to undergo senescence if cells experience mitogenic challenges or environmental stress. Most of the recent publications on AP4 point out that AP4 is involved directly in cell cycle progression, stemness, tumor initiation and progression, and metastasis, and it remains to be determined whether elevated levels of AP4 in tumors are due to increased expression of c-Myc or other causes. In the future, more work is needed to clarify how the levels of AP4 can affect its functions and its interaction with other transcription factors, such as AP1 and c-Myc. In addition, more efforts will be needed to elucidate more interacting partners of AP4 and their effects on AP4 function, as well as to how AP4/protein complexes orchestrate the diverse array of AP4 functions in both physiological and pathological contexts.
